# Race disparities in mortality by breast cancer from 2000 to 2017 in São Paulo, Brazil: a population-based retrospective study

**DOI:** 10.1186/s12885-021-08735-2

**Published:** 2021-09-07

**Authors:** Ana Cláudia Marcelino, Bruno Gozzi, Cássio Cardoso-Filho, Helymar Machado, Luiz Carlos Zeferino, Diama Bhadra Vale

**Affiliations:** Obstetrics and Gynecology Department, State University of Campinas, Rua Alexander Fleming 101, Campinas, SP CEP 13083-881 Brazil

**Keywords:** Breast neoplasms, Mortality, Race/ethnicity, Racial disparities, Black women, Healthcare disparities

## Abstract

**Background:**

In Brazil, inequalities in access may interfere with cancer care. This study aimed to evaluate the influence of race on breast cancer mortality in the state of São Paulo, from 2000 to 2017, contextualizing with other causes of death.

**Methods:**

A population-based retrospective study using mortality rates, age and race as variables. Information on deaths was collected from the Ministry of Health Information System. Only white and black categories were used. Mortality rates were age-adjusted by the standard method. For statistical analysis, linear regression was carried out.

**Results:**

There were 60,940 deaths registered as breast cancer deaths, 46,365 in white and 10,588 in black women. The mortality rates for 100,000 women in 2017 were 16.46 in white and 9.57 in black women, a trend to reduction in white (*p* = 0.002), and to increase in black women (*p* = 0.010). This effect was more significant for white women (*p* < 0.001). The trend to reduction was consistent in all age groups in white women, and the trend to increase was observed only in the 40–49 years group in black women. For ‘all-cancer causes’, the trend was to a reduction in white (*p* = 0.031) and to increase in black women (*p* < 0.001). For ‘ill-defined causes’ and ‘external causes’, the trend was to reduce both races (*p* < 0.001).

**Conclusion:**

The declared race influenced mortality rates due to breast cancer in São Paulo. The divergences observed between white and black women also were evident in all cancer causes of death, which may indicate inequities in access to highly complex health care in our setting.

## Background

Breast cancer is the main neoplasm that affects women in Brazil and worldwide, representing about 56 new cases and 13 deaths per 100,000 Brazilian women annually [1, 2]. It is a relevant public health problem due to the number of lives affected, the impact on potentials life-years lost, and the impact of the diagnosis and treatment on the health system. The incidence of breast cancer is increasing worldwide [3]. This increase is justified by the socioeconomic improvements, causing changes in women’s habits, increase exposure to risk factors, such as postmenopausal hormonal therapies and obesity, and the widespread use of mammography detecting indolent cancers [3–5].

In recent years, breast cancer mortality rates have decreased in high-income countries, increasing in low and middle-income countries [3, 6, 7]. Mortality from breast cancer is strongly influenced by stage and treatment [8]. The stage at diagnosis is influenced by women’s access and adherence to early diagnosis programs. When systematically applied to the target population, mammography can reduce mortality from breast cancer [9].

In South America and the Caribbean, the ratio between mortality and incidence is more significant than observed in Europe and North America [6]. In Latin America, 41% of women are diagnosed in stages III and IV, increasing incidence and mortality [10]. In Brazil, a country with significant regional inequalities, between 1980 and 2009 there was a trend to reduce mortality rates in the economically favoured Southeast region and to increase rates in the most impoverished Northeast region [11]. From 2001 to 2011, the Human Development Index significantly influenced a variation in mortality rates among Brazillian states [12]. Socioeconomic differences may explain those pieces of evidence.

Blacks and browns correspond to 55% of the Brazilian population and represent the most economically disadvantaged strata [13]. In addition to social marginalization, this group also faces structural racism that can hamper access to health services and compromise care quality. This population has a higher degree of illiteracy, lower income, uses health services less and is more dependent on the public health system [14]. They also have lower life expectancy and mortality rates due to external causes, drug use and homicides [14].

Mortality rates due to breast cancer increased in black and brown women in Brazil aged 50 or more between 2000 and 2010 [15]. A regional study from 2003 to 2005 found that black women with breast cancer were diagnosed at more advanced stages and had shorter survival than white women in the same stage of diagnosis: specific 10-year survival of 70% for white and 44% for black [16]. One hypothesis for the worst rates observed is that there could be variations in other causes of deaths, as improvements in the quality of registry in death certificates or decreases in other significant causes in the vulnerable population, such as “external” or violent causes.

To evaluate the influence of race on breast cancer, this work aims to demonstrate the evolution of mortality rates in São Paulo’s state as a function of race from 2000 to 2017. We also sought to contextualize breast cancer deaths with other causes of death. Age groups were also analyzed to identify where the differences are more significant. Finally, it is hoped that this study may support the planning of specific public policies for cancer control and the global health of black women.

## Methods

This is a population-based retrospective study using aggregated data on deaths from breast cancer among women living in the State of São Paulo, Southeast region of Brazil, whose death occurred between 2000 and 2017. Population data were obtained from the Brazilian Institute of Geography and Statistics (IBGE) [13]. The available variables age and race were analyzed. São Paulo’s population was stable in the period, not prone to movements fluxes.

Information on deaths was collected from the Ministry of Health through the Mortality Information System (MIS) [17]. In Brazil, death certificates are compulsory, and physicians fill forms. Health departments of each municipality collect the information and record it periodically in the online national registry system, using the 10th International Classification of Diseases (ICD-10) for registration. Completeness is high. In addition to extracting “breast cancer” data (ICD-10 C50), it was decided to extract data on other mortality categories to assist in the analysis of the hypothesis: “cancer” (filter ICD-10 C00-C97), “external causes” (filter ICD-10 S00- T99, V00-W99, Y00-Y99), “ill-defined causes” (filter “ill-defined causes”). Also, age groups were analysed to qualify the analysis.

The MIS provides filters for age and race. For the denominator in the race, were used the proportions of the race categories by age-group of the female population in the São Paulo state, according to the IBGE’s National Household Sample Survey (PNAD) [13]. The PNAD is a qualified census that permanently researches general characteristics of the population, education, work, income and housing, having the household as its research unit. The PNAD interviewers use the self-declared race for registration. When PNAD race data were not available (years 2000, 2010 and 2017), a projection was made by time trends. The IBGE and MIS categorize race into five classes: white, black, brown, yellow and indigenous. According to the proportion of the total population, the “ignored” race data were redistributed among the five categories. In Brazil, blacks and browns have the same African origin, brown indicating miscegenation of black with white people (European colones or immigrants). In this study we included only the white and black/brown categories (from now on grouped as “black”). There is a tendency to combine them as a single category by those who promtes racial debates, since both groups show similar socioeconomic indications of discrimination.

Deaths in children and women under 20 were discarded due to the high chance of corresponding to errors, and those “ignored” were redistributed by age groups (0.02%). It was also redistributed those “ignored” race (4.33%). The crude rates were calculated by the quotient between the total number of deaths and the population at risk for every 100,000 women. The adjusted rates per year were calculated considering the standard population of the World Health Organization of 2000 [18]. Then, the information on deaths by race versus *the* population’s proportion was crossed to calculate the crude mortality rates by race and then age-adjusted rates.

For statistical analysis of the difference in rates by race and age was used linear regression analysis, with a significance level of 5%. Three regression coefficients were used: B1, B2 and B3. B1 the increase or decrease over the years (the slope or year effect); B2 the difference in level between one series and another (the race effect); and B3 the difference between the slopes of the different series (the race vs year interaction term).

This study is part of the research project funded by FAPESP (2017/21908–1) and was approved by the Unicamp Research and Ethics Committee, registered at ‘Plataforma Brasil’ under the number CAAE 89399018.2.0000.5404. The Committee waived the need for the consent form due to the retrospective nature of the study and by the aggregated mode of data used.

## Results

**Fig. 1:**
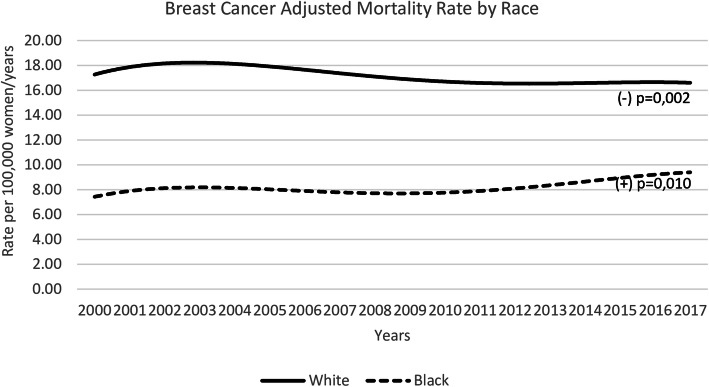
Breast cancer mortality rates in São Paulo State, Brazil, from 2000 to 2017, as a function of race. Legend: *P*-value refers to linear regression year-effect. (+) indicates trend to increase and (−) trend to decrease rates

In the State of São Paulo, from 2000 to 2017, there were 60,940 deaths registered as breast cancer deaths, 46,365 in white and 10,588 in black women. The figures of the adjusted mortality rates by years, race and age groups, are displayed in Table 1. Figure 1 shows the age-adjusted mortality rates over the years according to race. The rates for every 100,000 women varied from 19.1 in 2005 to 16.0 in 2014 in white (decrease of 16.2%) and from 7.0 in 2006 to 9.6 in 2017 in black (increase of 37.1%). Over the years it was observed a significant trend to reduction in white women mortality rates [B1 = (−)0.10; *p* = 0.002], and a significant trend to increase in black women mortality rates [B1 = (+)0.07; *p* = 0.010]. This effect was more significant for white than for black women [white women with a greater inclination to fall: B3 = (−)0.18; *p* < 0.001].

**Table 1:** Women breast cancer adjusted mortality rates in São Paulo, Brazil, by race and age-groups, from 2000 to 2017

Years	40 to 49 years		50 to 59 years		60 to 69 years		70 to 79 years		Total	
	White	Black	White	Black	White	Black	White	Black	White	Black
**2000**	2.6	1.7	3.9	1.9	3.9	1.7	3.5	1.0	17.1	7.4
**2001**	2.7	1.7	4.2	2.4	4.3	1.6	3.6	1.0	18.1	7.9
**2002**	2.6	1.4	4.6	2.3	4.4	2.0	3.2	0.9	18.4	7.9
**2003**	2.7	1.7	4.2	2.3	4.0	1.6	3.4	1.0	17.8	8.3
**2004**	2.7	1.8	4.2	2.3	4.3	1.7	3.3	1.4	17.9	8.6
**2005**	2.8	1.9	4.7	2.0	4.4	2.1	3.5	1.2	19.1	8.2
**2006**	2.4	1.4	3.7	2.1	4.1	1.6	3.5	0.8	16.8	7.0
**2007**	2.4	1.5	3.8	2.1	3.8	1.6	3.2	1.5	16.7	8.0
**2008**	2.7	1.6	4.0	1.9	4.0	1.6	3.3	1.2	17.6	7.4
**2009**	2.5	1.5	3.9	2.1	3.9	1.5	3.1	1.1	16.9	7.6
**2010**	2.4	1.6	3.9	2.4	4.0	1.7	3.2	1.3	16.7	8.4
**2011**	2.5	1.7	3.8	2.2	4.1	1.7	3.2	0.8	17.2	7.8
**2012**	2.4	1.8	3.8	2.2	3.9	1.6	2.9	1.1	16.3	8.2
**2013**	2.4	1.7	3.7	2.3	3.8	1.7	3.4	1.1	16.5	8.1
**2014**	2.5	2.1	3.6	2.4	3.7	2.0	3.0	1.2	16.0	9.3
**2015**	2.4	1.8	3.7	2.4	3.8	1.7	3.3	0.9	16.8	8.4
**2016**	2.7	2.1	3.7	2.2	3.9	1.9	3.3	1.2	17.0	9.1
**2017**	2.4	1.8	3.6	2.6	4.0	2.1	3.0	1.4	16.5	9.6
**B1 (p)**	−0.01 (0.032)	0.02 (0.018)	−0.05 (< 0.001)	0.02 (0.066)	0.02 (0.012)	0.01 (0.214)	−0.02 (0.004)	0.01 (0.435)	−0.10 (0.002)	0.07 (0.010)
**B2 (p)**	1.14 (< 0.001)		2.24 (< 0.001)		2.57 (< 0.001)		2.42 (< 0.001)		10.50 (< 0.001)	
**B3 (p)**	−0.03 (0.001)		−0.06 (< 0.001)		− 0.03 (0.006)		−0.03 (0.011)		− 0.18 (< 0.001)	

*p p*-value; (−) indicates a tendency towards a reduction in rates. Statistics by linear regression: B1, the slope or year effect; B2, the race effect; B3, the race vs year interaction term

**Fig. 2:**
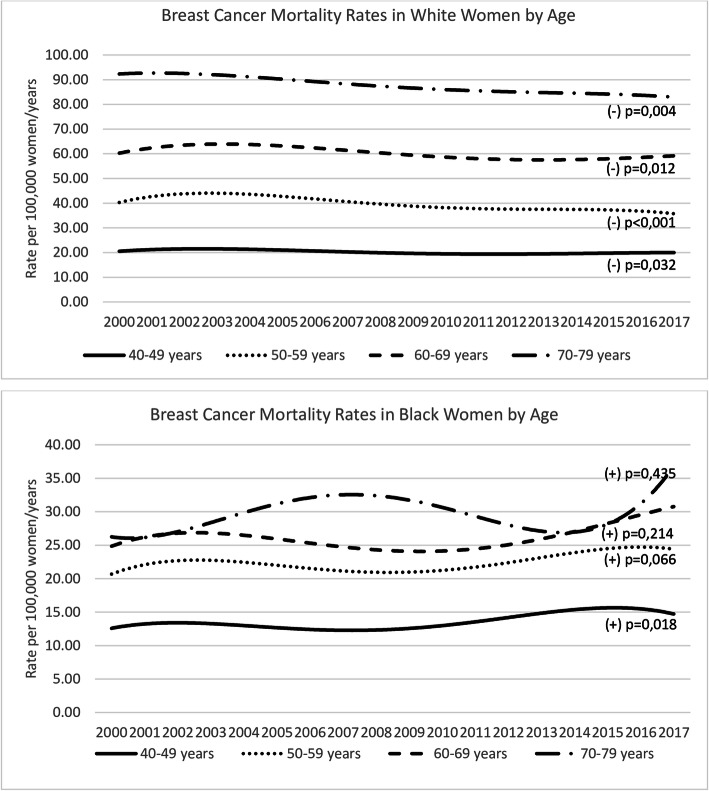
Breast cancer mortality rates in São Paulo State, Brazil, from 2000 to 2017, by age-groups, as a function of race. Legend: *P*-value refers to linear regression year-effect. (+) indicates trend to increase and (−) trend to decrease rates

The evaluation of variations in rates according to age-groups by race can be seen in Fig. 2. For women aged 40–49 years, mortality tended to decrease in white and to increase in black women [B1 = (−)0.01; *p* = 0.032 and B1 = (+)0.02; *p* = 0.018, respectively]. For the age-groups 50–59 years, 60–69 years and 70–79 years, the trend observed in white women was to a reduction in mortality in all age-groups [B1 = (−)0.05; *p* < 0.001, B1 = (−)0.02; *p* = 0.012 and B1 = (−)0.02; *p* = 0.004, respectively]. In black women, there was no significant variation for these age-groups [B1 = (+)0.02; *p* = 0.066, B1 = (+)0.01; *p* = 0.214 and B1 = (+)0.01; *p* = 0.435, respectively].

**Fig. 3:**
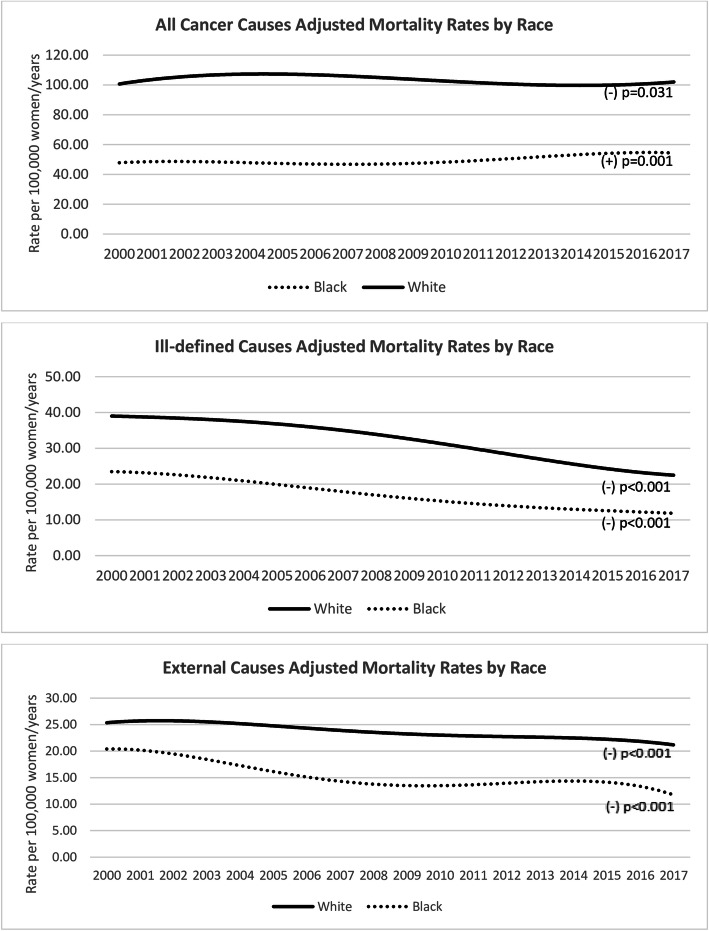
Breast cancer mortality rates in São Paulo State, Brazil, from 2000 to 2017, by causes of death and race. Legend: *P*-value refers to year-effect linear regression. (+) indicates trend to increase and (−) trend to decrease rates

The figures of the adjusted mortality rates by years and the causes of death are displayed in Table 2. The analysis of other causes of death according to race is presented in Fig. 3. In the category ‘all-cancer causes’, the trend was to a reduction in mortality rates in white women [B1 = (−)0.34; *p* = 0.031] and an increase in black women [B1 = (+)0.42; *p* < 0.001]. This effect was more significant for the reduction among white women [B3 = (−)0.75; *p* < 0.001]. For “ill-defined causes”, the trend was in a decrease among white and black women [B1 = (−)1.06; *p* < 0.001 and B1 = (−)0.75; *p* < 0.001], respectively. For ‘external causes’, there was a reduction over the years for both races [B1 = (−)0.26; *P* < 0.001 in white and B1 = (−)0.43; *P* < 0.001black women], with an explicit difference on the races (B2 = 6.84; *P* < 0.001).

**Table 2:** Adjusted women mortality rates in São Paulo, Brazil, by race and causes of death, from 2000 to 2017

Year	Breast Cancer		All cancer causes		Ill-defined causes		External causes	
	White	Black	White	Black	White	Black	White	Black
**2000**	17.1	7.4	100.4	47.3	37.9	23.1	25.3	20.8
**2001**	18.1	7.9	104.0	49.9	40.1	23.4	25.9	18.9
**2002**	18.4	7.9	104.8	47.7	38.9	22.5	25.3	20.1
**2003**	17.8	8.3	108.3	47.2	37.9	22.7	24.8	18.7
**2004**	17.9	8.6	106.6	50.2	38.0	21.4	26.3	17.4
**2005**	19.1	8.2	111.3	47.2	35.7	18.3	26.5	17.4
**2006**	16.8	7.0	101.7	45.4	35.0	18.8	22.8	14.3
**2007**	16.7	8.0	103.3	48.4	34.7	17.6	22.4	13.0
**2008**	17.6	7.4	107.7	45.0	33.5	16.6	24.2	13.0
**2009**	16.9	7.6	105.7	46.6	33.4	16.7	23.5	14.0
**2010**	16.7	8.4	101.0	50.0	32.1	16.1	22.4	14.8
**2011**	17.2	7.8	103.7	49.5	32.1	14.2	23.6	13.6
**2012**	16.3	8.2	100.7	50.7	27.5	14.1	23.7	14.0
**2013**	16.5	8.1	99.2	50.9	25.5	12.9	22.2	13.7
**2014**	16.0	9.2	97.7	54.7	24.6	13.3	22.3	15.0
**2015**	16.8	8.4	100.5	54.1	24.0	11.9	21.8	13.6
**2016**	17.0	9.1	101.6	52.7	25,0	12.3	21.9	12.9
**2017**	16.5	9.6	101.6	55.5	21.9	12.0	21.4	12.2
**B1 (p)**	−0.10 (0.002)	0.07 (0.010)	−0.34 (0.031)	0.42 (< 0.001)	−1.06 (< 0.001)	−0.75 (< 0.001)	−0.26 (< 0.001)	−0.43 (< 0.001)
**B2 (p)**	10.50 (< 0.001)		60.10 (< 0.001)		17.65 (< 0.001)		6.84 (< 0.001)	
**B3 (p)**	−0.18 (< 0.001)		− 0.75 (< 0.001)		− 0.31 (< 0.001)		0.17 (0.046)	

*p p*-value; (−) indicates a tendency towards a reduction in rates. Statistics by linear regression: B1, the slope or year effect; B2, the race effect; B3, the race vs year interaction term

## Discussion

In this study of assessment of breast cancer mortality rates from 2000 to 2017 in women from São Paulo, Brazil, it was observed that, while in white women the rates tend to a reduction, in black women the rates tend to increase. The same trends were observed in cancer mortality rates in general. The woman’s age in the year of death did not influence this result, as the death records’ qualification and the reduction in deaths due to external or violent causes.

The reduction in breast cancer mortality is the result of improved access to early diagnosis and timely treatment. The therapeutic evolution, especially the development of new drugs and specific therapies, is considered the main factor that has impacted breast cancer mortality in recent decades [8, 19]. Trastuzumab, for example, is available in the universal health care system since 2013 in São Paulo state, so women carrying the HER2/neu protein would have a better prognosis [19].

However, inequity in access to therapeutic modalities can influence population rates in different ways. The trend observed in the reduction of mortality in white women as opposed to the increase in rates in black women indicates the evident influence of race on this issue in the Brazilian population.

The incidence rates of breast cancer are increasing in Brazil and worldwide [3]. Therefore, without adequate intervention, mortality rates are also expected to increase. The increase in mortality rates among black women may reflect delay in diagnosis, difficulty in accessing highly complex services for treatment or biological variations. In North American women, rates in black women are 21% lower for the hormonal receptor (HR) and HER2 positive, 29% higher in HR negative and HER2 positive, and 93% higher for triple-negative subtypes [20]. This data may justify the rough differences observed among race, but not the opposite trends observed in the study.

The increase in cancer mortality rates in general observed in this study suggests that access to treatment should be a significant factor influencing these results. Delays in surgical treatment due to race are reported in the USA [21]. Also, racial minorities are more likely to receive more non-standardized treatments (out of standard protocols), such as less indication for surgical treatment, fewer sentinel lymph node biopsies and reconstructions, and less access to adjuvant treatment [21–24]. There is a greater chance of delaying its onset regarding chemotherapy and hormone therapy, which can lead to significant differences in survival [21, 25]. In Brazil, more significant delays have been reported in populations with lower income, less education and non-white ethnic groups [26].

A possible explanation for these evidence is that socioeconomic variables are influencing the race factor. However, it is necessary to consider a potential implicit bias in the health professional behaviour when a difference in the preference for a given social group would negatively impact communication, clinical investigation and decision in the treatment of vulnerable patients [22]. In Brazil, an assessment of satisfaction with health care and hospitalization observed 12.2% of non-satisfied white patients and 17.4% among blacks and browns patients [14]. The probability of a black or brown person looking for a health service and not being seen is twice as high as a white person [14].

In general, international literature does not support the hypothesis of a biological plausibility that would justify these observed differences. A population-based study in the USA showed that the black population is twice as susceptible to death as the white population in tumours that express hormone receptors in the first 2 years after diagnosis, with no difference between the triple-negative types. The disparities persisted after accounting for factors as stage, tumour characteristics, and body mass index. However, black/white disparities persisted after accounting for those factors. Authors suggest that incomplete or delayed receipt of treatment may have influenced the results [27]. Another study points out that despite hormonal subtype, socioeconomic status or access to health services, racial disparities in diagnosis, treatment and survival still persist [23].

The incidence of hormone receptor-negative tumours is higher in black than in white women [23, 28, 29]. Certain genetic factors may be associated with this fact, such as more frequent mutations in BRCA and others. However, the real functional impact of these on the incidence and progression of triple-negative cancers in this population is still unknown [24]. In the specific case of tumours with negative hormone receptors, it is known that the delay in diagnosis and referral can compromise the effectiveness of the treatment [23].

In this study, data on stage and molecular subtypes were not available. It is known that more aggressive tumours tend to affect younger populations [30, 31]. In women aged 40 to 49, mortality decreased significantly among white women and increased significantly among black women. It is not possible to determine whether tumours in black women were more aggressive than those in white women. One explanation to have not observed increased trends in older women is the low number of cases at the sub-groups. Still, this data also reinforces the hypothesis of less access to treatment faced by black women. Survival studies can elucidate this issue.

The hypothesis that an improvement may have influenced mortality rates in the quality of filling out death certificates among black women in the period was investigated. The analysis of mortality rates due to ill-defined causes revealed that the tendency towards reduction was observed for both white and black women, with a more critical fall among white women, which could have influenced the results in an inverse way to the hypothesis made. Another interesting fact is that in the period, there was a significant reduction in mortality from external causes in the population in general, more significant among white women, which should not have influenced the results either.

This is a population-based study in a region that concentrates about 1/5 of the Brazillian population, representing other large urban centres in middle-income countries. São Paulo state focuses most of the high-quality care in Brazil, public and private, so the results found in this study must be highlighted when extrapolating to other regions where health care is not so developed. However, it is also important to note that the data from the death registry information system in the State of São Paulo is considered the best in the country. In this way, we believe that this is the best information obtained for the analyses to be carried out.

The study’s main limitations are the lack of data on the stage and molecular subtypes of breast cancers. Also, we did not have data in incidence to interpret the study results better. However, we believe that access to treatment, which would mainly affect mortality, is parallel to access to diagnosis, which would mainly affect incidence. In this sense, once treatment care would be improved, diagnosis also would be better. No significant cultural behaviour change was observed in habits in the period to influence risk factors. We believe that future survival analyzes from population-based cancer registries can support the evidence generated in this study.

## Conclusion

The declared race influenced mortality rates due to breast cancer in São Paulo. The divergences observed between white and black women also were evident in all cancer causes of death, which may indicate inequities in access to highly complex health care in our setting.

## Data Availability

The datasets analyzed during the current study are available in the SIM repository: http://tabnet.datasus.gov.br/cgi/deftohtm.exe?sim/cnv/obt10sp.def and the IBGE repository: https://www.ibge.gov.br/estatisticas/sociais/populacao.html. The datasets generated during and/or analyzed during the current study are available from the corresponding author on reasonable request.

## Abbreviations

B1: The slope or year effect
B2: The difference in level between one series and another (the race effect)
B3: The difference between the slopes of the different series (the race vs year interaction term).
IBGE: Brazilian Institute of Geography and Statistics
ICD-10: 10th International Classification of Diseases
HR: Hormonal Receptor
MIS: Mortality Information System
PNAD: National Household Sample Survey
